# Efficient expression of nuclear transgenes in the green alga *Chlamydomonas*: synthesis of an HIV antigen and development of a new selectable marker

**DOI:** 10.1007/s11103-015-0425-8

**Published:** 2016-01-08

**Authors:** Rouhollah Barahimipour, Juliane Neupert, Ralph Bock

**Affiliations:** Max-Planck-Institut für Molekulare Pflanzenphysiologie, Am Mühlenberg 1, 14476 Potsdam-Golm, Germany

**Keywords:** *Chlamydomonas reinhardtii*, Molecular farming, HIV, Antigen, Selectable marker gene, Transformation

## Abstract

The unicellular green alga *Chlamydomonas reinhardtii* has become an invaluable model system in plant biology. There is also considerable interest in developing this microalga into an efficient production platform for biofuels, pharmaceuticals, green chemicals and industrial enzymes. However, the production of foreign proteins in the nucleocytosolic compartment of *Chlamydomonas* is greatly hampered by the inefficiency of transgene expression from the nuclear genome. We have recently addressed this limitation by isolating mutant algal strains that permit high-level transgene expression and by determining the contributions of GC content and codon usage to gene expression efficiency. Here we have applied these new tools and explored the potential of *Chlamydomonas* to produce a recombinant biopharmaceutical, the HIV antigen P24. We show that a codon-optimized *P24* gene variant introduced into our algal expression strains give rise to recombinant protein accumulation levels of up to 0.25 % of the total cellular protein. Moreover, in combination with an expression strain, a resynthesized *nptII* gene becomes a highly efficient selectable marker gene that facilitates the selection of transgenic algal clones at high frequency. By establishing simple principles of successful transgene expression, our data open up new possibilities for biotechnological research in *Chlamydomonas*.

## Introduction

The unicellular green alga *Chlamydomonas* *reinhardtii* is widely used as a model organism for research on fundamental questions in cell and molecular biology, including chloroplast biology, photosynthesis, light perception and signaling as well as flagellar function and tactic movements (Harris 2001; Merchant et al. 2007). In its vegetative phase, *Chlamydomonas* is a haploid organism and can be grown photoautotrophically, mixotrophically or heterotrophically (using acetate as the sole source of organic carbon; Harris 1989). Triggered by environmental cues, two vegetative cells of opposite mating types (mt+ and mt−) can differentiate into gametes and engage in sexual reproduction. All three genomes of the alga (in the nucleus, the plastid and the mitochondria) are completely sequenced (Merchant et al. 2007) and can be stably transformed (Kindle et al. 1991; Remacle et al. 2006; Neupert et al. 2012).

The ease with which *Chlamydomonas* can be cultured in large volumes and the ease with which it can be genetically engineered have also made the alga a preferred model organism in biotechnological research, especially for metabolic engineering, biofuel production and the synthesis of proteinaceous biopharmaceuticals and industrial enzymes, an area commonly referred to as molecular farming (Scaife et al. 2015; Scranton et al. 2015). However, harnessing the full potential of *Chlamydomonas* in biotechnology turned out to be challenging because of severe problems with (i) the expression of nuclear transgenes to reasonable levels (Fuhrmann et al. 1999; Schroda et al. 2000), and (ii) epigenetic transgene inactivation mechanisms that cause loss of expression with time (Yamasaki et al. 2008). Although introduction of transgenes into the nuclear genome of the alga is straightforward, the isolation of transgenic strains that express the foreign gene of interest to detectable levels, in many cases, has turned out to present an unsurmountable challenge (Mussgnug 2015). Identification of promoters suitable to drive strong transcription of heterologous genes (Fischer and Rochaix 2001), construction of hybrid promoters (Schroda et al. 2000) and resynthesis of the coding region of the transgene to adjust its codon usage to that of the *Chlamydomonas* nuclear genome (Fuhrmann et al. 1999, 2004; Shao and Bock 2008), has led to some improvements, but did not provide a general solution to the transgene expression problem. Recently, we described a genetic screen that facilitates the selection of mutant algal strains that express introduced reporter genes to high levels (Neupert et al. 2009). Two mutant strains (UVM4 and UVM11) were isolated from a UV mutagenesis experiment (Neupert et al. 2009) and have quickly become widely used as versatile tools for transgene expression and subcellular localization analyses (e.g., Karcher et al. 2009; Zäuner et al. 2012; Bohne et al. 2013; Lauersen et al. 2013, 2015; reviewed, e.g., in Jinkerson and Jonikas 2015).

In addition to promoter choice and the genetic constitution of the recipient strain, the efficiency of transgene expression is influenced by the properties of the coding region. The *Chlamydomonas* nuclear genome has an unusually high GC content (Merchant et al. 2007) and genes with low GC content or strongly deviating codon usage are known to be expressed only very poorly or not at all (Fuhrmann et al. 1999; Shao and Bock 2008; Barahimipour et al. 2015). Although GC content and codon usage are connected (in that both are influenced by selective pressure on nucleotide composition of the genome), their relative contributions to expression efficiency are experimentally separable. Expressing *YFP* gene variants that differ in GC content and/or codon usage, we have recently demonstrated that codon usage, rather than GC content, is the most important determinant of transgene expression efficiency in *Chlamydomonas reinhardtii* (Barahimipour et al. 2015). Together with the availability of strong promoters (Schroda et al. 2000; Fischer and Rochaix 2001) and algal expression strains that permit high-level transgene expression (Neupert et al. 2009; Karcher et al. 2009), this finding raises hopes that we now have the most important tools required to optimize foreign gene expression in *Chlamydomonas* and elevate it to levels that are comparable to other systems (such as yeasts and seed plants). This would make the alga competitive with other production platforms in biotechnology and open up new possibilities for its commercial use.

Here, we have applied the knowledge gained about nuclear transgene expression and explored the potential of *Chlamydomonas* for the expression of two biotechnologically relevant genes: the gene for the HIV-1 antigen P24, a likely indispensable component of any future AIDS vaccine, and the kanamycin resistance gene *nptII*, a widely used selectable marker gene that, however, does not work well for *Chlamydomonas* (Hall et al. 1993; Bingham et al. 1989). Originally isolated from the *Escherichia coli* transposon Tn5, it has quickly become widely used as a selectable marker for transformation experiments in both prokaryotes and eukaryotes. It also represents the by far most frequently used marker for nuclear transformation in seed plants and also works as a selectable marker for chloroplast transformation in tobacco (*Nicotiana tabacum*; Elghabi et al. 2011; Carrer et al. 1993). However, attempts to develop *nptII* as a marker for nuclear transformation in *Chlamydomonas* have remained largely fruitless. Although introduction of *nptII* as a passenger gene along with a different selectable marker (nitrate reductase; Hall et al. 1993) resulted in clones that displayed low-level kanamycin resistance, direct selection for kanamycin resistance was largely unsuccessful and, in one published study, produced only a single exceptional transformation event (Bingham et al. 1989).

Since the beginning of the acquired immune deficiency syndrome (AIDS) epidemic, 78 million people worldwide have been infected with the human immunodeficiency virus (HIV, mainly with variant HIV-1) and 39 million people have died of the disease (http://www.who.int/gho/hiv/en/). Globally, approximately 35 million people were living with the virus in 2013, and a large number of these infections are located in developing countries. A safe, effective and affordable vaccine that reduces transmission of HIV-1 or, alternatively, prevents disease progression is desperately needed. Unfortunately, despite more than 30 years of intense research efforts, there is still no effective AIDS vaccine on the horizon. The complex biology of the infection process and the high mutation rate of the virus (Trkola 2004) make it likely that a multi-component vaccine comprising several antigenic polypeptides of HIV-1 will be required to invoke broad and potent immunity. Provided that suitable candidate antigens for recombinant subunit vaccines can be identified, crop plants or edible algae (such as *Chlamydomonas reinhardtii*) would provide the ideal production platform to cheaply produce large quantities of an AIDS vaccine that can be administered orally and is stable even without an uninterrupted cooling chain.

The HIV-1 protein P24 (or p24) forms the conical core of HIV-1 viral particles. P24 represents the largest cleavage product of the precursor polyprotein encoded by the *gag* gene of the retrovirus. P24 is the target of T cell immune responses in both primary infected and chronically infected AIDS patients. Also, capsid proteins, such as P24, are preferred candidates for vaccine components, because of their high conservation due to structural and functional constraints, thus facilitating the targeting of specific epitopes that lie within conserved domains of the viral particle. As P24 is expected to be a crucial component of any future AIDS vaccine (Obregon et al. 2006), enormous efforts have been undertaken to develop expression strategies for the high-yield and cost-effective production of recombinant P24 protein (Meyers et al. 2008). Proof-of-concept studies in tobacco have shown that the protein can be expressed from the nuclear genome of plants (Zhang et al. 2002; Obregon et al. 2006) and, much more efficiently, from the plastid genome (Zhou et al. 2008; McCabe et al. 2008; Shanmugabalaji et al. 2013). Importantly, several immunogenicity studies with tobacco-derived P24 protein demonstrated elicitation of T cell responses in experimental animals (e.g., Meyers et al. 2008; Gonzalez-Rabade et al. 2011).

In this study, we have compared *P24* and *nptII* gene variants that encode the identical amino acid sequence but differ in codon usage, and tested them in our expression strain UVM11 and a wild type-like strain of *Chlamydomonas reinhardtii*. We demonstrate that fully codon optimized gene versions introduced into our algal expression strains allow high-level expression of the P24 antigen and turn the *nptII* gene into an efficient selectable marker gene for *Chlamydomonas*. Our work reported here establishes straightforward rules for successful transgene expression in *Chlamydomonas*, and opens up new applications in algal biotechnology.

## Materials and methods

### Algal strains and culture conditions

The *Chlamydomonas reinhardtii* cell wall-deficient strains Elow47 and UVM11 (Neupert et al. 2009) were used for all transformation experiments. Both strains are derived from the arginine auxotrophic strain *cw15*–*302* (cwd *mt*+ arg7). Strain Elow47 was generated by co-transformation of *cw15*–*302* with the *CRY1*–*1* emetine resistance gene and the *ARG7* gene providing arginine prototrophy. Strain UVM11 was obtained by UV mutagenesis of Elow47 followed by selection for high transgene expression (Neupert et al. 2009). Algal cells were cultivated mixotrophically in liquid Tris-acetate-phosphate (TAP) medium (Harris, 1989) or on agar-solidified TAP medium at 22 °C under continuous illumination (light intensity: 50–100 µE m^−2^ s^−1^), unless otherwise stated.

### Construction of transformation vectors

Three gene variants of *P24* (encoding the conical core subunit of HIV-1 viral particles) that differ in their GC content and codon usage were generated as follows: NdeI and EcoRI recognition sequences containing a start codon and a stop codon, respectively, were introduced upstream and downstream of the coding regions of all *P24* gene variants. Variant *P24w* is identical to the wild-type *P24* sequence (nucleotides 508–1200 of HIV-1, isolate BH10, Gene Bank accession number: M15654.1). Variant *CrP24* was codon optimized for the nuclear genome of *Chlamydomonas* (using the codon frequency table of the Kazusa database: http://www.kazusa.or.jp/codon/) and chemically synthesized (GeneCust). Both versions were cloned as NdeI/EcoRI restriction fragments into pJR38 (Neupert et al. 2009) digested with the same enzymes, giving rise to plasmids pRMB18 and pRMB19, respectively. *CpP24*, an AT-rich lowly codon-adapted variant (codon optimized for the tobacco chloroplast genome) was amplified from plastid transformation vector pZF1 (Zhou et al. 2008) using primers CpP24NdeIf (5′-AAGCCCAT **ATG**CCTATTGTACAAAATATTCAAGG-3′) and CpP24EcoRIr (5′-TGCCAGAATTC **TTA**GAGTACTCTAGCTTTATG-3′; restriction sites underlined, start and stop codons indicated in bold). The PCR amplicon was cloned into the pCR2.1-TOPO^®^ TA vector and, after sequence confirmation, the *CpP24* gene was excised as NdeI/EcoRI restriction fragment and ligated into the similarly digested plasmid pJR38, generating transformation vector pRMB20.

Two gene variants of neomycin phosphotransferase II gene *nptII*, *EcnptII* and *CrnptII*, with identical amino acid sequence but different GC content and codon usage were analyzed. The AT-rich *EcnptII* sequence (originally from *E. coli* transposon Tn5) was amplified from plastid transformation vector pRB96 (Wurbs et al. 2007) using primers NptIINdeIf (5′-CAAGCCCAT **ATG**GAACAAGATGGATTG-3′) and NptIIEcoRIr (5′-AGAATTC **TTA**GAAGAACTCGTCAAGAAGGCG-3′; restriction sites underlined, start and stop codons indicated in bold) that introduce NdeI and EcoRI restriction sites at the 5′ and 3′ ends of the gene, respectively. The PCR product was cloned into vector pCR2.1-TOPO^®^ TA (Invitrogen). After sequence confirmation, the NdeI/EcoRI restriction fragment was cloned into the similarly digested plasmid pJR38 (Neupert et al. 2009), resulting in transformation vector pRMB28. The gene sequence of the *CrnptII* variant was codon optimized according to the preferred codon usage in the *Chlamydomonas* nuclear genome using the codon frequency table of the Kazusa database (http://www.kazusa.or.jp/codon/) and then chemically synthesized (GeneCust, Dudelange, Luxembourg). Unique NdeI and EcoRI restriction sites at the 5′ and 3′ ends of the gene, respectively, were used for cloning into the similarly digested plasmid pJR38, generating transformation vector pRMB27.

### Transformation of *Chlamydomonas reinhardtii*

Nuclear transformation of the Elow47 and UVM11 strains of *Chlamydomonas reinhardtii* was performed using the glass bead method and following published protocols (Kindle 1990; Neupert et al. 2012). 1 µg of plasmid DNA linearized with ScaI or NaeI was used for transformation of the *P24* variants into the algal genome. Transformants were selected on TAP medium containing 10 µg mL^−1^ paromomycin. Transformation with the *nptII* gene variants was performed with 250 ng of gel-eluted (NucleoSpin^®^ Gel and PCR Clean-up kit, Macherey–Nagel, Düren, Germany) XhoI/XbaI restriction fragment containing the *nptII* coding region and the *PSAD* promoter and terminator sequences. The *aphVIII* cassette under the control of the *HSP70/RBCS2* promoter was eluted after digestion of pJR38 with restriction enzymes KpnI and XhoI. Transformants were selected on TAP medium supplemented with 25–200 µg mL^−1^ kanamycin.

### DNA isolation, Southern blot analysis and PCR

Total genomic DNA from *Chlamydomonas* was extracted according to published protocols (Schroda et al. 2001). 100 ng DNA were used as template for PCR assays and 10 µg were used for Southern blot analyses.

To identify transformants that have the complete *P24* transformation cassette integrated into their nuclear genome (i.e., the selectable marker gene and the entire transgene of interest), PCR assays were conducted using primer pairs that amplify sequences upstream and downstream of the gene of interest. Primers PPsaDrev (5′ CGAGCCCTTCGAACAGCCAGGCCG 3′) and M13for (5′ GTAAAACGACGGCCAGT 3′) amplified the 5′ end of the *PsaD* promoter upstream of the coding region of the transgene of interest (380 bp amplicon), and primers APHVIII.rev (5′ CCTCAGAAGAACTCGTCCAACAGCC 3′) and APHVIII.fw (5′ GGAGGATCTGGACGAGGAGCGGAAG 3′) amplified the 3′ end of the *aphVIII* selectable marker gene (360 bp amplicon). Transformed algal strains yielding both PCR products were selected as positive clones.

For Southern blot analysis, samples of 10 µg DNA were digested with the appropriate restriction enzymes, separated in a 1 % agarose gel, and transferred onto a Hybond™ N^+^ nylon membrane (GE Healthcare) by capillary blotting. An [α-^32^P]dCTP-labeled probe was produced by random priming (Megaprime™ DNA labeling system, GE Healthcare) using the complete open reading frame of *CrP24* as template. Hybridization took place at 65 °C according to standard protocols.

### RNA extraction and northern blot analysis

Total RNA was isolated from algal cultures using the Direct-Zol^TM^ RNA MiniPrep kit (Zymo Research) and following the manufacturer’s protocol. Samples of 10 µg RNA were separated in 1.2 % agarose gels containing 2 % formaldehyde and then transferred onto Hybond™ N^+^ nylon membranes (GE Healthcare) by capillary blotting. Hybridization was performed at 65 °C using [α-^32^P]dCTP-labeled probes (GE Healthcare) that were produced by random priming (Megaprime™ DNA labeling system, GE Healthcare). Restriction fragments covering the entire reading frame of the transgene were excised with NdeI and EcoRI from the corresponding transformation vectors and used as templates for probe generation. A 1:1 mixture of *CrnptII* and *EcnptII* fragments was used to produce a probe capable of detecting both transcripts with equal sensitivity.

### Protein extraction and immunoblot analyses

Total protein was extracted using a phenol-based extraction method (Cahoon et al. 1992). Extraction was performed by resuspension of the cell pellet in extraction buffer [0.7 M sucrose, 0.5 M Tris/HCl, 50 mM EDTA, 0.1 M KCl pH 9.4, 2 % 2-mercaptoethanol and 1× protease inhibitor cocktail cOmplete, EDTA-free (Roche, Darmstadt, Germany)]. An equal volume of phenol (Roti^®^-Phenol, Roth, Germany) was added, the sample was mixed thoroughly and centrifuged at 15,000×*g* for 10 min at 4 °C. The supernatant was transferred to a new tube and mixed with five volumes of 0.1 M NH_4_OAc in methanol. Proteins were precipitated overnight at −20 °C, and pelleted by centrifugation at 15,000×*g* and 4 °C for 5 min. Pellets were washed with 0.1 M NH_4_OAc in methanol, air-dried and resuspended in 1 % SDS at 60 °C for 3 min. Protein concentration of the extracts was determined with the BCA assay kit (Pierce, Rockford, IL,USA). Samples of 40 µg protein were separated by electrophoresis in 15 % SDS-PAA gels and subsequently transferred onto PVDF membranes (GE Healthcare, UK) using a standard transfer buffer (192 mM glycine, 25 mM Tris/HCl, pH 8.3). Blocking was performed with either 5 % BSA for detection of P24, or 2.5 % BSA and 2.5 % milk powder for detection of the NptII protein at room temperature for 1 h. Immunodetection of NptII was done with a 1:1000 dilution of rabbit anti-NptII primary antibody (Sigma) and a 1:50,000 dilution of anti-rabbit HRP-conjugated secondary antibody (Agrisera). The P24 protein was detected with a 1:1000 dilution of a monoclonal mouse anti-P24 primary antibody (Abcam) and a 1:5000 dilution of anti-mouse HRP-conjugated antibody (Agrisera). Hybridization signals were visualized by the ECL^TM^ Prime detection system (GE Healthcare).

### Antibiotic resistance tests

To compare the phenotypic resistance of *aphVIII* and *CrnptII* transformants to different antibiotics, nine independently transformed clones per transgene (initially selected on TAP medium containing 25 µg mL^−1^ kanamycin) were randomly chosen and maintained on agar-solidified TAP medium without antibiotics. Cultures grown in antibiotic-free liquid TAP medium were used for drop tests on agar-solidified TAP medium containing either 2.5–25 µg mL^−1^ G418 (G418 disulphate salt solution, Sigma-Aldrich), 5–50 µg mL^−1^ paromomycin (paromomycin sulphate, Duchefa Biochemie B.V.) or 25-200 µg mL^−1^ kanamycin (kanamycin monosulphate monohydrate, Duchefa Biochemie B.V.) under constant light of 50 µE m^−2^ s^−1^. To determine the level of resistance of *EcnptII* and *CrnptII* transformants to different concentrations of kanamycin (0–200 µg mL^−1^), additional drop tests were performed with dilution series of cultures of the 10 independent transformed strains (initially selected on 50 µg mL^−1^ kanamycin) that had been characterized by northern blot and immunoblot analyses.

## Results

### Design of *P24* gene variants for expression in *Chlamydomonas*

**Fig. 1:**
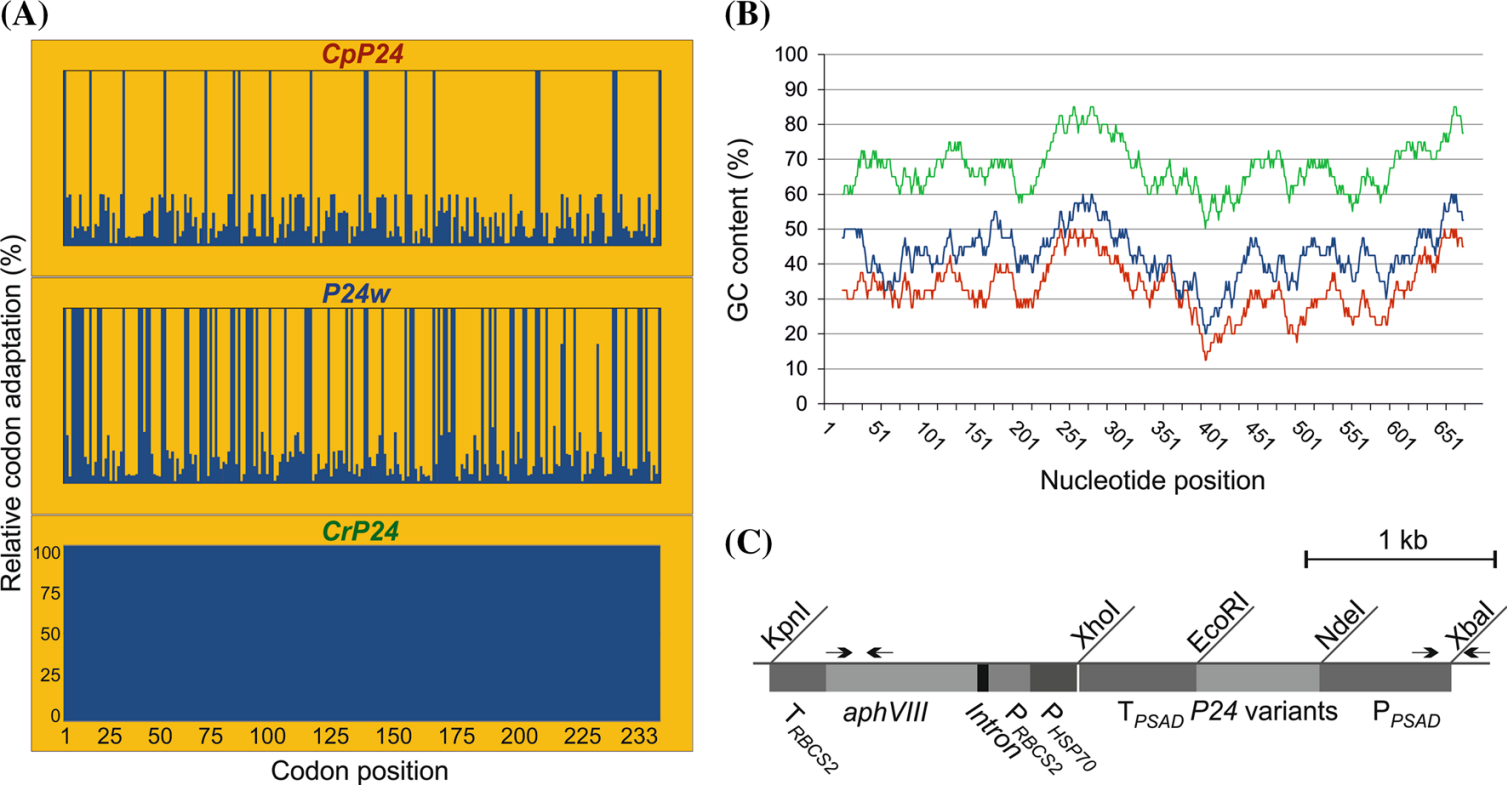
HIV-1 *P24* gene variants and physical map of the expression vector used for algal nuclear transformation. **a** Relative codon adaptation (RCA) of the different *P24* gene variants compared to the nuclear genome of *Chlamydomonas reinhardtii*. *Blue bars* indicate the relative adaptation (in %) of each codon in the reading frames of the three synthetic gene variants. The x-axis indicates the codon numbers within the gene (cf. Table 1). Variant *CrP24* contains the most frequently used synonymous codon for all amino acids. **b** GC content (in %) and its distribution over the reading frames of the three *P24* gene variants. The values were determined in a sliding window of 40 bp. *CrP24* is indicated in green, *P24w* in blue and the chloroplast-optimized variant *CpP24* in *red* (see *color code* in *panel a*). The least adapted variant (*CpP24*) has the lowest GC content and the fully codon-optimized gene version (*CrP24*) has the highest GC content (cf. Table 1). **c** Physical map of the transformation vector used for expression of the three *P24* gene variants in *Chlamydomonas reinhardtii*. All variants were cloned into the same vector and are driven by identical expression elements. The *arrows* indicate the binding sites of primer pairs used for PCR analysis of transformed algal strains. The different *P24* coding regions were inserted into an expression cassette derived from the *Chlamydomonas reinhardtii* *PSAD* locus (Fischer and Rochaix 2001) using the restriction sites NdeI and EcoRI (P_*PSAD*_: *PSAD* promoter; T_*PSAD*_: *PSAD* terminator). The paromomycin resistance gene *aphVIII* serves as selectable marker and is driven by the fused promoters from the *HSP70A* gene (P_*HSP70*_) and the *RBCS2* gene (P_*RBCS2*_) of *C. reinhardtii*

To identify factors involved in expression of the HIV antigen P24 in *Chlamydomonas reinhardtii*, we synthesized three variants of the *P24* gene that encode the identical amino acid sequence but differ in codon usage and GC content (Fig. 1a, b; Table 1): (i) the wild-type sequence from HIV-1 subsequently referred to us *P24w*, (ii) a version with the codon usage optimized for the AT-rich chloroplast genome (*CpP24*), and (iii) a version with the codon usage optimized for the GC-rich nuclear genome of *Chlamydomonas* (*CrP24*). Comparing codon usage and GC content, the *CpP24* gene variant is expected to have the most unfavorable gene sequence for expression from the nuclear genome of *Chlamydomonas*, whereas the wild-type variant *P24w* has more triplets that are frequently used in *Chlamydomonas* and also a higher GC content than *CpP24* (Fig. 1a, b). The three gene versions were cloned into the same expression cassette, inserted into the same transformation vector (Fig. 1c) and transformed into two different strains of *Chlamydomonas* by glass bead-assisted DNA delivery: the expression strain UVM11 isolated from a UV mutagenesis-based genetic screen for algal strains with improved expression properties, and the wild type-like control strain Elow47 (Neupert et al. 2009). This resulted in altogether six sets of transgenic algal clones that were compared with each other with respect to their efficiency of expressing the P24 antigen.

**Table 1:** Properties of the different *P24* and *nptII* gene variants used in this study

Gene variant	Codon optimization	Length (bp)	GC content (%)	Overall relative codon adaptation (%)
*CpP24*	Chloroplast	699	33	21
*P24w*	HIV	699	43	38
*CrP24*	Nucleus	699	68	100
*EcnptII*	*E. coli*	792	59	57.8
*CrnptII*	Nucleus	792	73	100

The variants of each transgene have the same size and the identical amino acid sequence but vary in their GC content and codon usage. The table gives the genome for which the gene variant was optimized, the length of the coding region, the GC content and the RCA relative to the nuclear genome of *Chlamydomonas reinhardtii*. See text for details

### mRNA and protein accumulation from the different gene variants in wild type-like stains and expression strains

From each of the six transformation experiments, 24 clones were randomly picked and integration of the *P24* expression cassette was verified by PCR assays (Fig. 1c; see “Materials and methods” section). On average, 58 % of the UVM11 transformants and 51 % of the Elow47 transformants were found to contain the complete *P24* cassette.

**Fig. 2:**
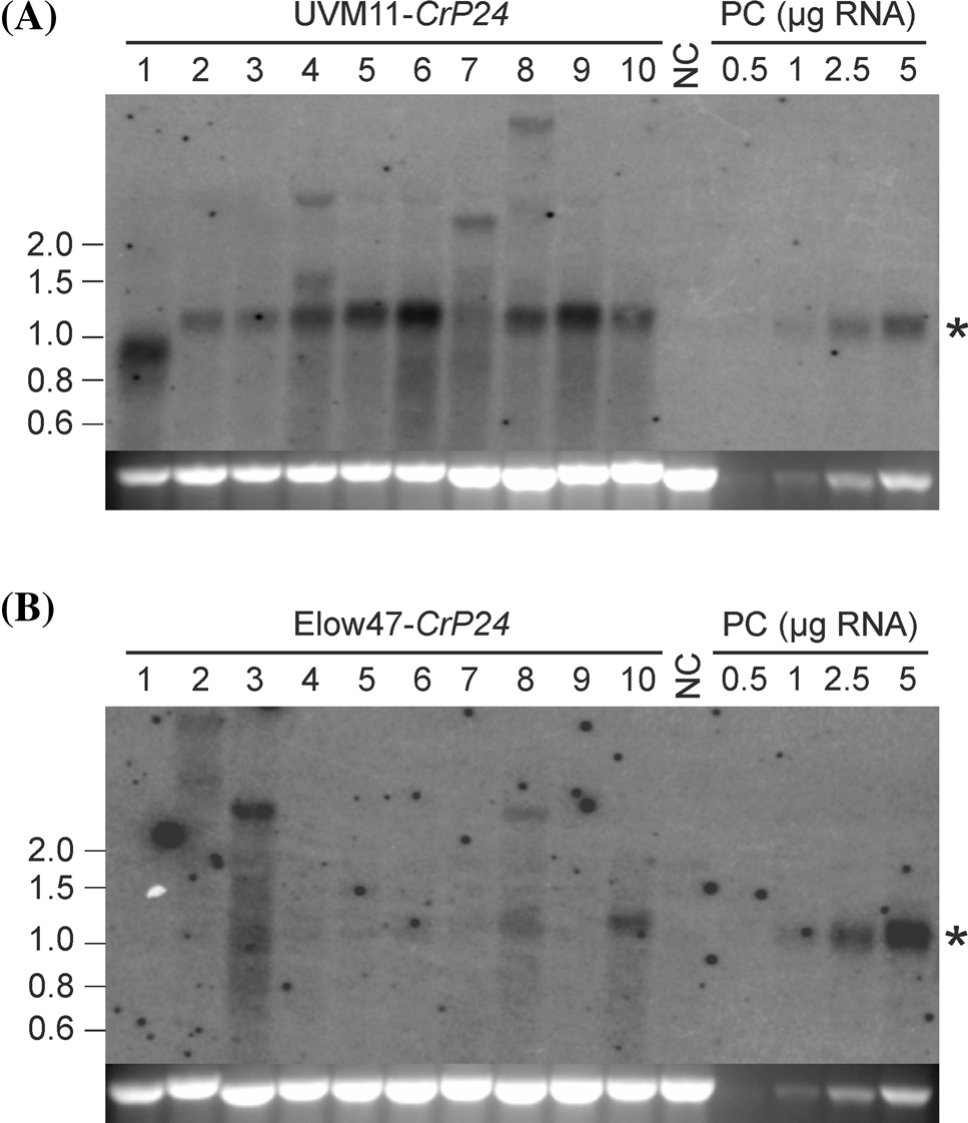
Analysis of *CrP24* mRNA accumulation in transformants of strains UVM11 and Elow47. Ten independent transformants harboring the complete *CrP24* cassette (based on PCR assays; see “Materials and methods” section) were selected randomly for each strain. 10 µg of total RNA was used for northern blot analysis. Transformant number five of strain UVM11 was selected as a standard for relative quantitation, and a dilution series of total RNA (0.5, 1, 2.5, 5 µg) of this line was loaded in all blots as a positive control (PC). The untransformed strain was used as negative control (NC). *Asterisks* indicate the expected transcript size of 1.1 kb. The ethidium bromide-stained gel prior to blotting is shown below each blot and serves as loading control. The whole reading frame of *CrP24* was used as hybridization probe. Marker band sizes are given in kb at the *left*. Additional transcripts of larger size may originate from multicopy insertions in tandem and/or from insertion into endogenous genes in the genome. **a** Northern blot analysis of UVM11 transformants. Transformed clone number 1 shows a slightly shorter *CrP24* transcript, presumably because of a small deletion or truncation. **b** Northern blot analysis of Elow47 transformants

For each experiment, ten clones that tested positive in both PCR reactions were subsequently assayed for *P24* expression. Interestingly, none of the analyzed *CpP24* and *P24w* transformants showed detectable levels of P24 accumulation (see below), neither in the wild-type background nor in the UVM11 expression strain. Therefore, these strains were not further analyzed. By contrast, the fully codon-optimized *CrP24* variant conferred strong mRNA accumulation in all tested transformants of the expression strain UVM11 and also resulted in detectable *P24* mRNA accumulation in a few transformants of strain Elow47 (Fig. 2).

**Fig. 3:**
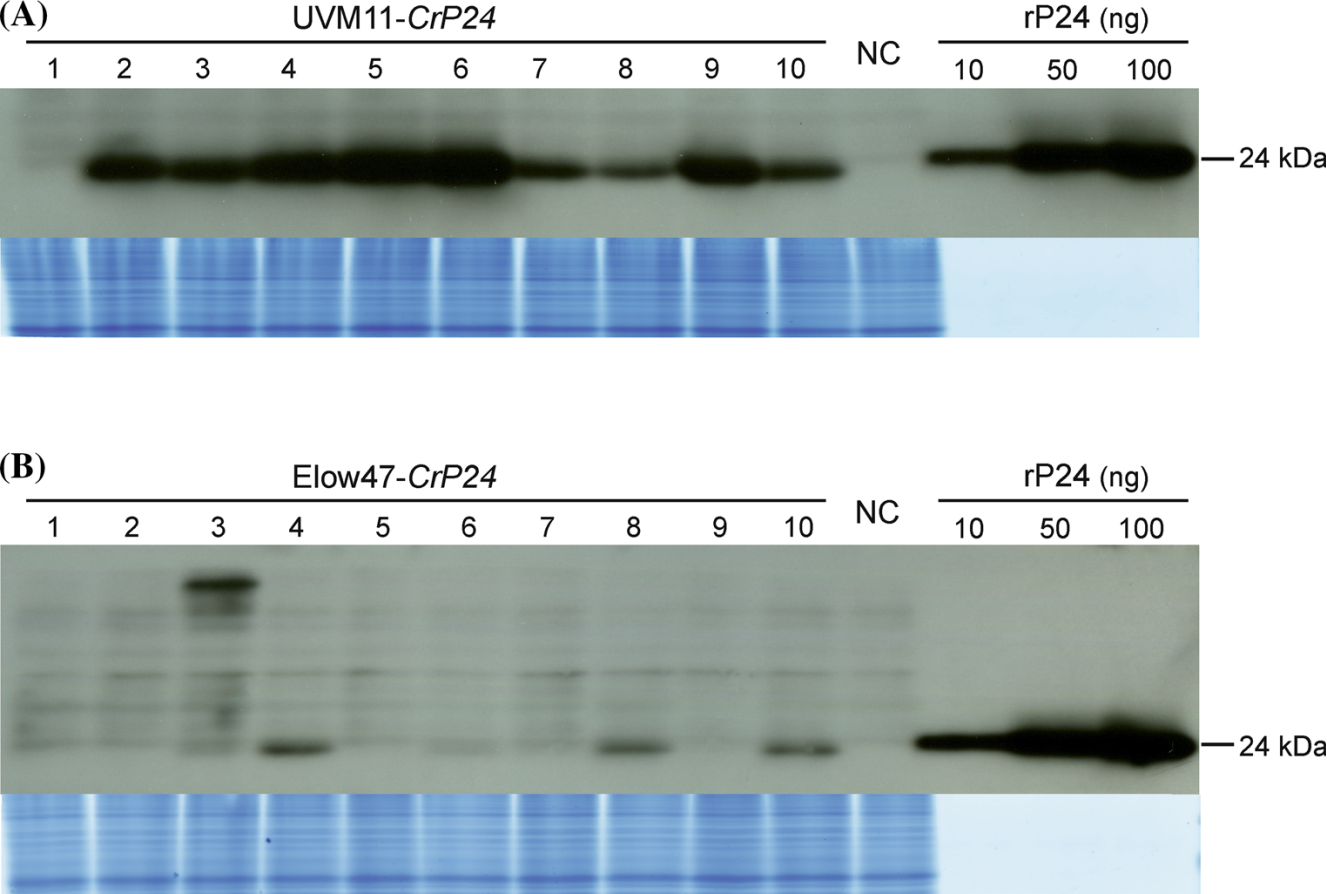
Immunoblot analysis of P24 protein accumulation in strains UVM11 and Elow47 transformed with the *P24* gene variant that was codon optimized for the *Chlamydomonas* nuclear genome. The same ten *CrP24* transformants from each strain that had been analyzed by northern blots (Fig. 2) were tested for P24 accumulation. 40 µg of total protein from each transformant were separated by SDS-PAGE. The untransformed strain was used as negative control (NC). A dilution series of recombinant His-tagged P24 protein (rP24) was loaded for semiquantitative analysis of P24 accumulation levels. The small size difference between the recombinant protein and the protein expressed in algal cells is due to the His-tag. The upper part of the gel was stained with Coomassie and served as loading control (shown below each blot). **a** Immunoblot analysis of UVM11 transformants. Note that transformed clone number 1 that accumulates a truncated *CrP24* transcript (Fig. 2a), is the only strain that does not accumulate the P24 protein. The maximum P24 accumulation level is approximately 0.25 % of total cellular protein (strain 6). **b** Immunoblot analysis of Elow47 transformants. Clone number 3 shows a larger-than-expected protein band (of approximately 36 kDa), consistent with accumulation of a larger mRNA (Fig. 2b). The larger protein may originate from in-frame fusion with an endogenous gene

To determine the extent to which mRNA accumulation correlates with protein accumulation, western blot experiments were conducted. To facilitate quantitation of P24 accumulation, a dilution series of recombinantly expressed P24 was included. No P24 protein could be detected in any of the *CpP24* and *P24w* transformants (not shown). Three of the *CrP24* transformants in strain Elow47 showed low levels of P24 protein accumulation (Fig. 3), among them the two clones that accumulated clearly detectable levels of the *P24* mRNA (cf. Fig. 2b). All transformed clones in strain UVM11 displayed strong P24 accumulation, with only moderate variation between transformants (Fig. 3). Overall, mRNA levels correlated well with protein levels in that the three UVM11 clones (numbers 5, 6 and 9; cf. Fig. 2a) that displayed higher mRNA levels than the others also showed the highest protein accumulation levels. In the best transformants, P24 accumulated to approximately 0.25 % of the total cellular protein of the alga, as determined by comparison with a dilution series of purified recombinant P24 protein (Fig. 3a).

**Fig. 4:**
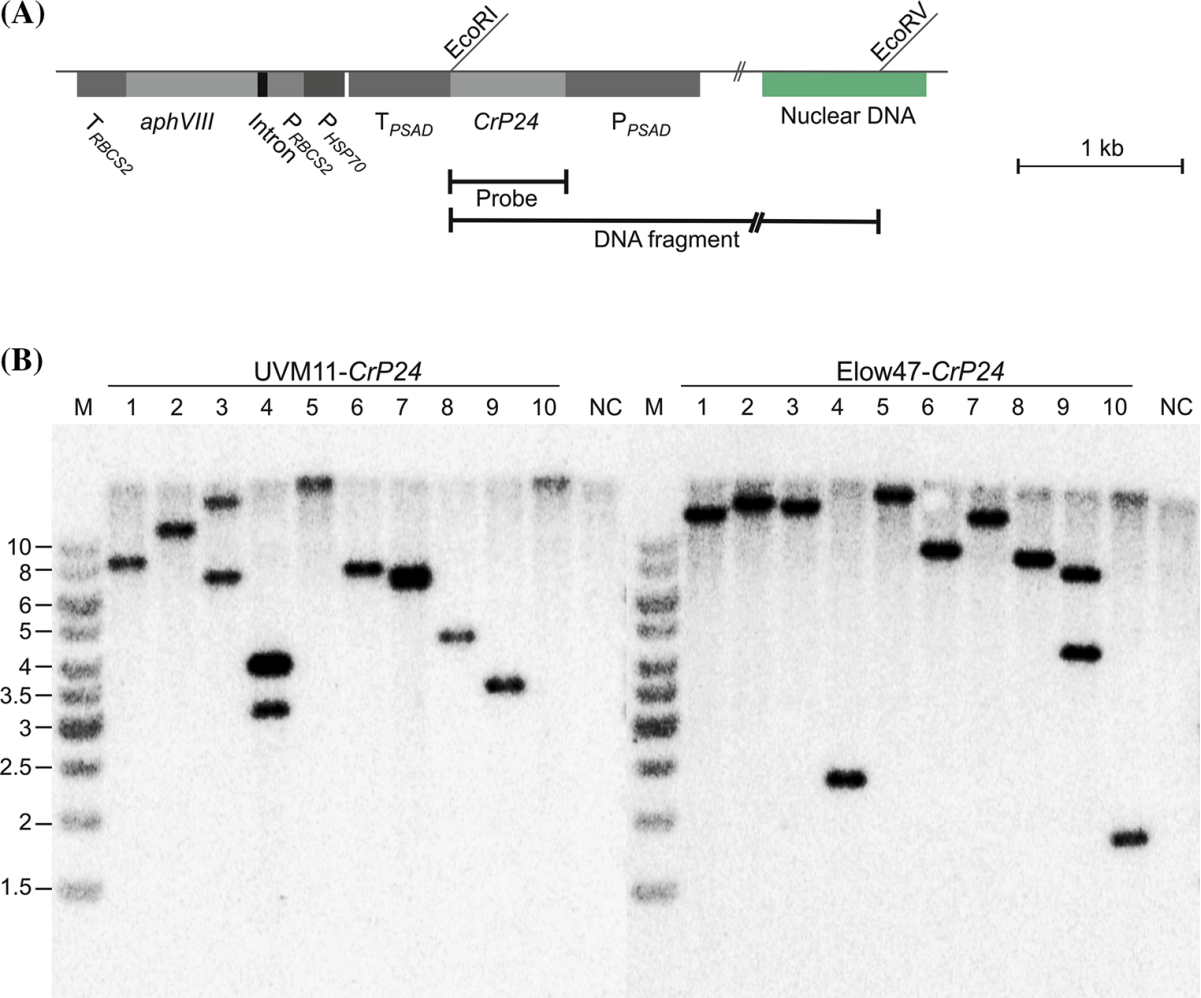
Southern blot analysis of *CrP24* transformants of strains UVM11 and Elow47. **a** Physical map of the transformation vector integrated into the *Chlamydomonas* nuclear genome. The EcoRI and EcoRV restriction sites used for RFLP analysis are indicated. The location of the EcoRV site in the flanking chromosomal DNA is hypothetical (and variable depending on the integration site of the transgenes). The hybridization probe (‘Probe’) and the restriction fragment it detects (‘DNA fragment’) are also indicated. *aphVIII*: paromomycin resistance gene (selectable marker); P_*HSP70*_: promoter from the *HSP70A* gene; P_*RBCS2*_: promoter from the *RBCS2* gene; P_*PSAD*_: promoter from the *PSAD* gene; T_*PSAD*_: terminator from the *PSAD* gene. **b** Southern blot analysis of ten randomly picked *CrP24* transformants of expression strain UVM11 (*left panel*) and ten randomly picked transformants of control strain Elow47 (*right panel*). Samples of 10 µg total DNA were digested with the restriction enzymes EcoRI and EcoRV and separated by agarose gel electrophoresis. DNA samples extracted from untransformed strains were used as negative control (NC) and digested with the same enzymes. The hybridization probe was generated by labeling a DNA fragment covering the entire coding region of *CrP24*. Fragment sizes of the molecular weight marker (M) are given at the left in kb. Note that the majority of the transformants harbors a single copy of the *CrP24* transgene

To assess whether or not expression correlated with transgene copy number in the nuclear genome of the alga, Southern blot experiments were performed. They revealed that most of the transformants harbor only a single transgene copy (Fig. 4). Importantly, the three best-performing transformants (UVM11 transformants 5, 6 and 9) all contain only a single copy of the *P24* cassette. Consistent with previous data (Barahimipour et al. 2015), these data establish that transgene copy number is not positively correlated with expression level. Instead, optimum expression is achieved when a fully codon-optimized transgene is expressed in the UVM11 expression strain.

### Introduction of variants of the kanamycin resistance gene *nptII* into *Chlamydomonas* cells

Having confirmed the importance of strain background and codon usage as the two key factors that determine transgene expression efficiency in the nucleus of *Chlamydomonas*, we reasoned that these principles should also be applicable to the development of new selectable marker genes for algal transformation. A number of selectable marker genes that work in nearly all organisms are very inefficient or do not work at all in *Chlamydomonas*. The kanamycin resistance gene *nptII* provides a case in point. Although being a nearly universally applicable marker for transformation in seed plants, attempts to develop *nptII* as a marker for nuclear transformation in *Chlamydomonas* have remained largely unsuccessful (Hall et al. 1993; Bingham et al. 1989).

**Fig. 5:**
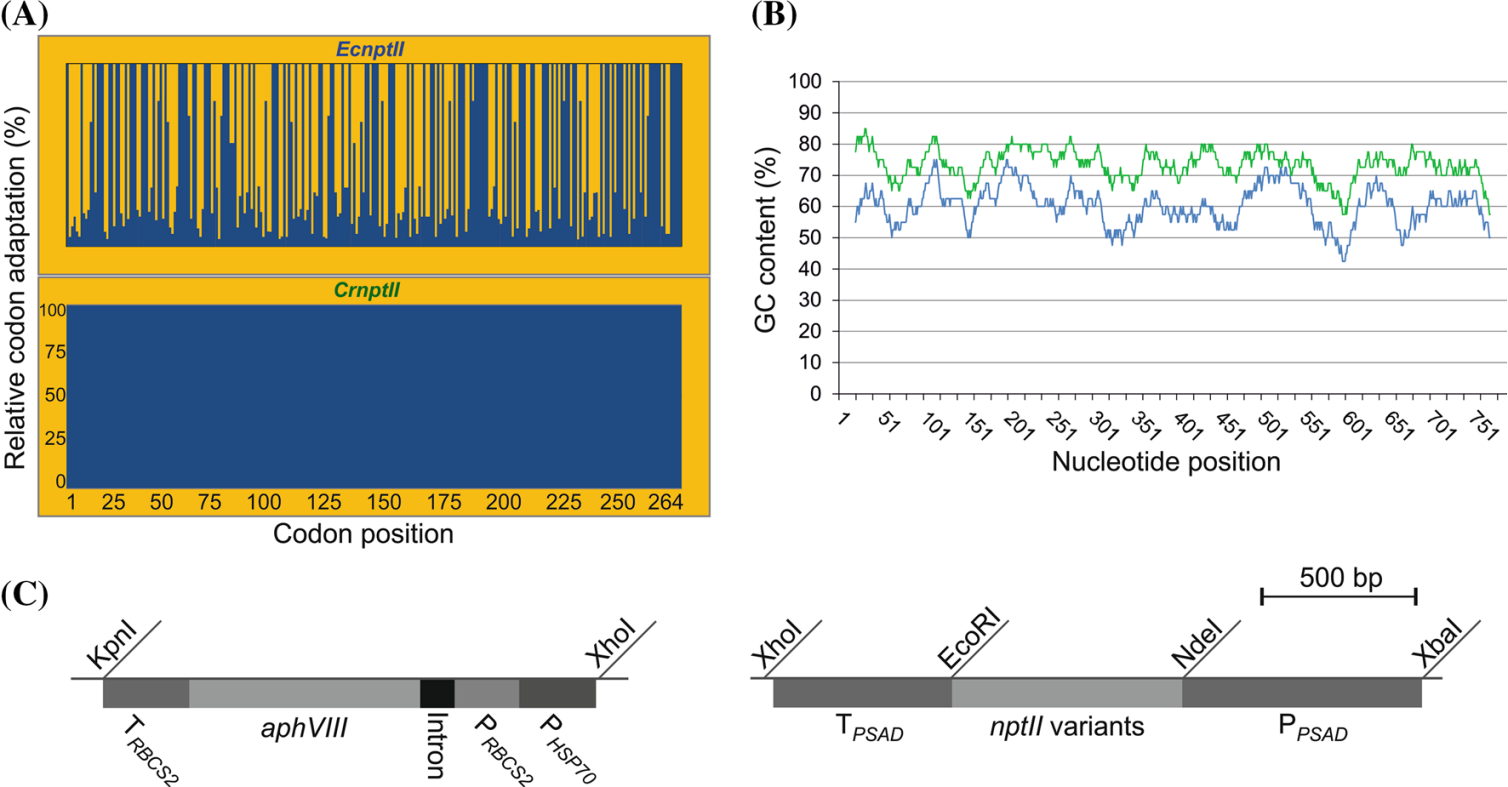
Codon usage, GC content and physical map of the expression vector used for transformation of *Chlamydomonas* with *nptII* gene variants and with *aphVIII*. **a** Relative codon adaptation (RCA) of the two *nptII* gene variants compared to the nuclear genome of *Chlamydomonas*. *Blue bars* indicate the relative adaptation (in %) of each codon in the reading frames of the two gene variants. The x-axis indicates the codon numbers within the gene. *CrnptII* contains the most frequently used synonymous codon for all amino acids. **b** GC content (in  %) and its distribution over the reading frames of the two *nptII* variants. The values were determined in a sliding window of 40 bp. *CrnptII* is indicated in *green* and the original (bacterial) *EcnptII* in *blue*. The fully codon-optimized *CrnptII* has a higher GC content. **c** Physical map of the transformation vectors used for expression of the paromomycin resistance gene *aphVIII* (*left panel*) and the two *nptII* gene variants (*right panel*) in *Chlamydomonas*. The *aphVIII* gene is driven by the fused promoters from the *HSP70A* gene (P_*HSP70*_) and the *RBCS2* gene (P_*RBCS2*_) of *C. reinhardtii*. The *nptII* coding regions were inserted into an expression cassette derived from the *Chlamydomonas PSAD* locus (Fischer and Rochaix 2001) using the restriction sites NdeI and EcoRI (P_*PSAD*_: *PSAD* promoter; T_*PSAD*_: *PSAD* terminator)

To test whether *nptII* can be turned into an efficient selectable marker gene for *Chlamydomonas*, we produced two gene variants and inserted them into identical expression cassettes for nuclear transformation (Fig. 5). Gene variant *EcnptII* represents the original *nptII* gene from the *E. coli* transposable element Tn5. It is relatively GC rich (59 %) and shows a relative codon adaptation (RCA; Fox and Erill 2010) of 57.8 %, a value much higher than that of the *CpP24* and the *P24w* gene versions (cf. Fig. 1; Table 1). The RCA represents a reference set-based index in which the codon with the highest frequency (fraction value) is set to 100 % relative adaptiveness and all other triplets for the same amino acid are scaled accordingly (by calculating their frequency of occurrence relative to the codon with the highest usage).The second *nptII* variant, *CrnptII*, was fully codon optimized for the preferred codon usage in the nuclear genome of *Chlamydomonas reinhardtii* and has a GC content of 73 % (Fig. 5a, b).

Using glass bead-assisted transformation, the two *nptII* variants were introduced into *Chlamydomonas* expression strain UVM11 and the wild type-like strain Elow47 (Neupert et al. 2009). Transformed clones were selected on medium containing a relatively low concentration of kanamycin (50 µg mL^−1^) to facilitate the selection of transformants also with the non-codon-optimized gene version in strain Elow47. In this way, four sets of transgenic algal clones were generated and subsequently compared with respect to their efficiency of expressing the *nptII* gene at the mRNA and protein levels.

### mRNA and protein accumulation from the two *nptII* gene variants in strains Elow47 and UVM11

**Fig. 6:**
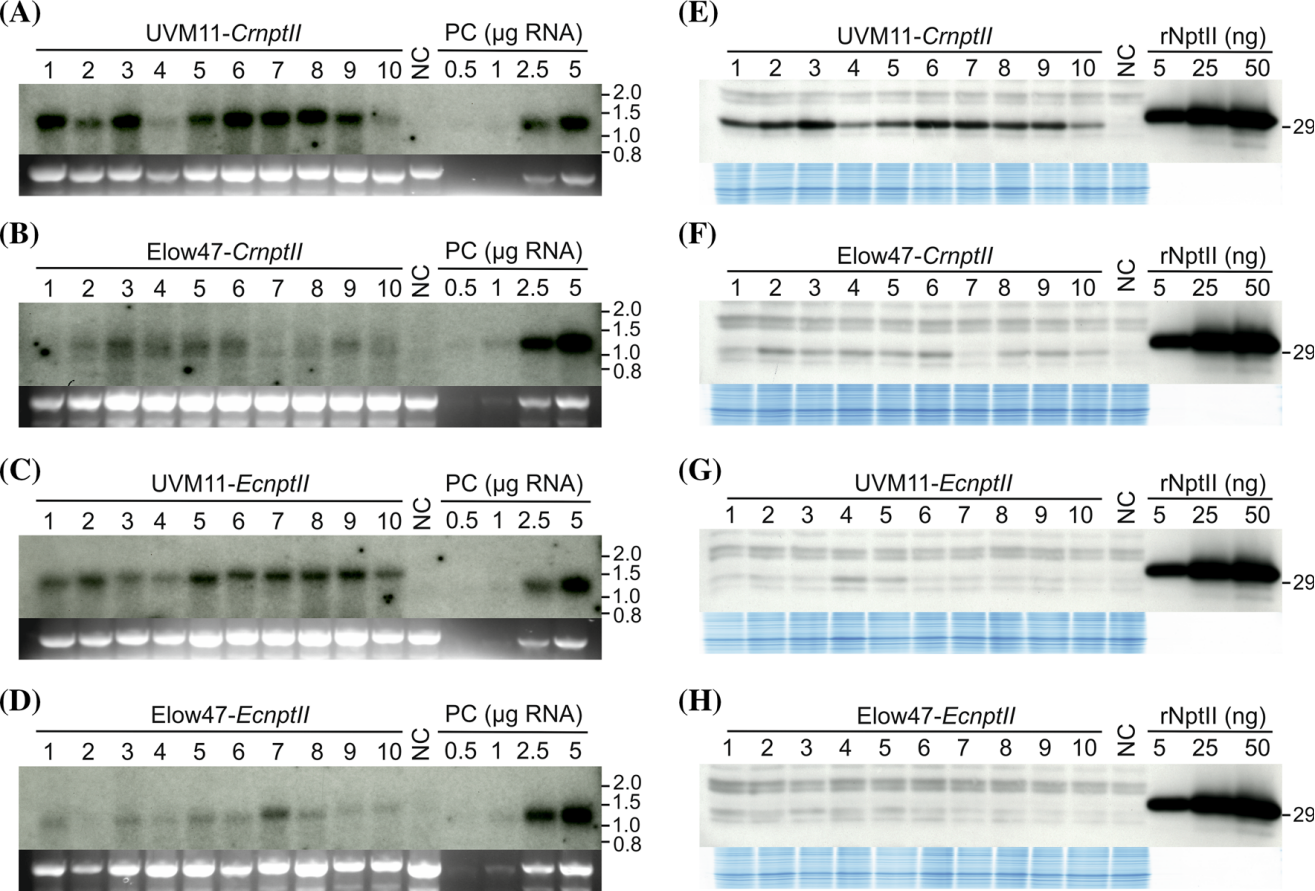
Comparison of *CrnptII* and *EcnptII* transcript accumulation in *Chlamydomonas* strains UVM11 and Elow47 by northern blot analysis (**a**–**d**), and protein accumulation levels conferred by the two *nptII* variants as determined by immunoblot analysis (**e**–**h**). All analyzed strains were selected on kanamycin (50 µg mL^−1^) and, therefore, are expected to express the *nptII* marker gene. For the northern blots (*panels a*–*d*), samples of 10 µg total RNA were electrophoretically separated in denaturing 1.2 % agarose gels. The gel blots were hybridized to a radiolabeled probe comprising a 1:1 mixture of the complete coding regions of both *nptII* variants. RNA samples isolated from the corresponding untransformed strains were used as negative control (NC). A dilution series (0.5, 1.0, 2.5 and 5.0 µg RNA) of an NptII-expressing algal clone (*CrnptII* transformant number 3 in strain UVM11) was loaded onto each gel as a positive control (PC) to facilitate comparison between blots. The band representing the 25S rRNA of the cytosolic 80S ribosome in the ethidium bromide-stained gel prior to blotting is shown below each blot as a loading control. Sizes of RNA marker bands are indicated in the right of each blot (in kb). The size of the *nptII* transcript is approximately 1.2 kb. For the immunoblots (panels e–h), samples of 40 µg total cellular protein were separated by SDS-PAGE and the same transformed strains were analyzed as in panel (a-d). A dilution series of recombinant NptII (rNptII) was loaded to facilitate semiquantitative analysis and comparison between blots. The size of the untagged protein expressed in *Chlamydomonas* is ~29 kDa, the slightly larger size of the rNptII is due to its His-tag. Protein samples of the untransformed strains were loaded as negative controls (NC). As a control for equal loading, the Coomassie-stained upper part of the gel is shown below each blot. **a** *nptII* mRNA accumulation in ten independent transgenic clones of expression strain UVM11 transformed with gene variant *CrnptII*. **b** *nptII* mRNA accumulation in ten independent clones of strain Elow47 transformed with gene variant *CrnptII*. **c** *nptII* mRNA accumulation in ten independent transgenic clones of expression strain UVM11 transformed with the *EcnptII* gene variant. **d** *nptII* mRNA accumulation in ten independent transgenic clones of strain Elow47 transformed with the *EcnptII* gene variant. **e** NptII protein accumulation in ten independent transgenic clones of expression strain UVM11 transformed with gene variant *CrnptII*. **f** NptII protein accumulation in ten independent clones of strain Elow47 transformed with gene variant *CrnptII*. **g** NptII protein accumulation in ten independent transgenic clones of expression strain UVM11 transformed with the *EcnptII* gene variant. Note that strains 4 and 5 show above-background expression of NptII, whereas in all other transformed clones, the signal is not stronger than that of the cross-reacting band of similar size in the NC. **h** NptII protein accumulation in ten independent transgenic clones of strain Elow47 transformed with the *EcnptII* gene variant

The four transformation experiments resulted in dramatically different transformation efficiencies, with the wild type-like strain Elow47 producing very low numbers of kanamycin-resistant clones, as expected (for quantitative analysis, see below). From each of the four transformation experiments, ten kanamycin-resistant clones were randomly picked and assayed for *nptII* mRNA accumulation and NptII protein abundance (Fig. 6). It is important to note that all analyzed transgenic clones were primarily selected for kanamycin resistance and, therefore, are expected to express the *nptII* transgene sufficiently well to confer resistance to 50 µg mL^−1^ kanamycin. Thus, different from our P24 expression experiments, transgenic clones that do not express the *nptII* transgene (as all analyzed transformants with non-codon optimized *P24* genes; see above) were not recovered.

Analysis of *nptII* mRNA accumulation revealed that the *nptII* transcript was detectable in nearly all selected clones (Fig. 6a–d), consistent with their kanamycin-resistant phenotype. However, there were significant differences between the four sets of transgenic algal clones in the average expression level of *nptII*. On average, mRNA accumulation was highest in UVM11 transformed with *CrnptII*, followed by UVM11 transformed with *EcnptII*, whereas transcript levels were low in Elow47 transformed with *CrnptII*, and lowest in Elow47 transformed with *EcnptII* (Fig. 6a–d). Expression of the *nptII* gene in all randomly selected UVM11 transformants confirms that the kanamycin selection does not produce a significant fraction of false positive clones (i.e., escapes or spontaneous resistance mutants), a conclusion that is in line with the absence of kanamycin-resistant colonies from control plates with untransformed wild-type cells.

When protein accumulation levels were determined by immunoblot analyses using an anti-NptII antibody, strong NptII accumulation was seen in UVM11 clones transformed with *CrnptII* (Fig. 6e). Significantly lower levels of NptII accumulated in Elow47 transformed with *CrnptII* and UVM11 transformed with *EcnptII*, whereas protein levels were below the detection limit in most Elow47 clones transformed with *EcnptII* (Fig. 6f–h). As observed with expression of the codon-optimized *P24* gene in UVM11, the NptII protein accumulation levels were nearly uniformly high in all tested UVM11-*CrnptII* clones (Fig. 6e).

In all analyzed UVM11 transformants, the kanamycin resistance phenotype remained stable over time and cultivation cycles in that the clones displayed unaltered drug resistance after half a year of growth under non-selective conditions.

### Transformation efficiencies with the two *nptII* gene variants in strains Elow47 and UVM11

**Fig. 7:**
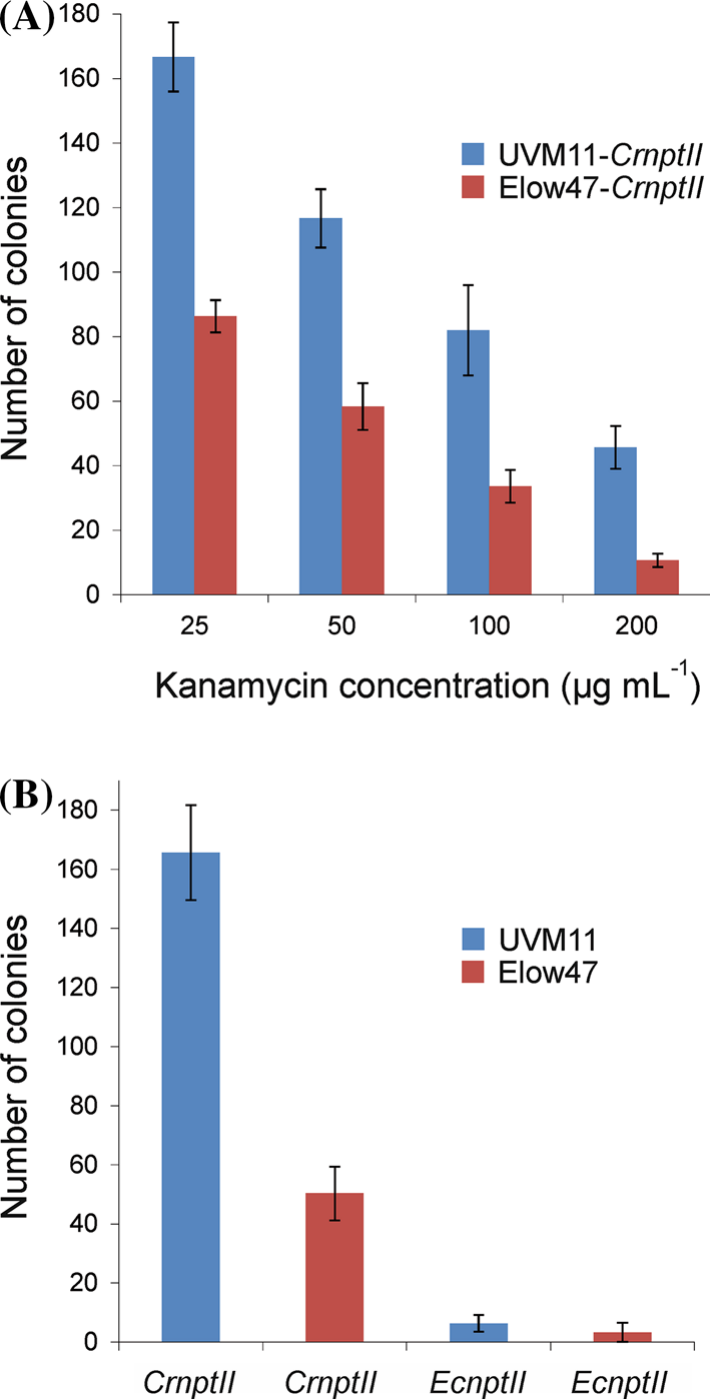
Transformation efficiencies obtained with the two *nptII* gene variants in *Chlamydomonas* strains UVM11 and Elow47. **a** Analysis of transformation efficiencies obtained with *CrnptII* in expression strain UVM11 and the corresponding wild type-like strain Elow47 at different concentrations of kanamycin. The strains were transformed with the *CrnptII* cassette and selection was performed on media containing different concentrations of kanamycin (25, 50, 100 and 200 µg mL^−1^). The number of kanamycin-resistant colonies was averaged from three independent transformation experiments. *Error bars* indicate the standard deviation. **b** Comparison of transformation efficiencies obtained with the two *nptII* variants in strains UVM11 and Elow47. Algal cells were transformed with either the *CrnptII* or the *EcnptII* cassette and selected on medium containing 50 µg mL^−1^ kanamycin. The number of resistant colonies was averaged from three independent transformation experiments (scored 6 days after transformation). *Error bars* indicate the standard deviation

When the transformation experiments with the two *nptII* gene variants and the two algal strains were performed, we noticed that the transformation efficiencies were vastly different. To verify this observation and quantify the differences between constructs and strains, the transformation experiments were repeated and the transformation frequencies were determined from three sets of transformation experiments. The data revealed that, indeed, the transformation efficiencies were greatly different. When strains UVM11 and Elow47 were transformed with the codon-optimized *CrnptII* gene version, the transformation frequency was approximately twice as high with UVM11 upon selection for low levels of kanamycin resistance (25 µg mL^−1^). Interestingly, the difference between the two strains became greater upon selection on higher antibiotic concentrations, reaching an approximately fivefold higher transformation frequency in UVM11 at 200 µg mL^−1^ kanamycin (Fig. 7a). This observation is likely explained by the higher *nptII* expression levels in UVM11 which allow efficient antibiotic detoxification even at very high concentrations of kanamycin where most Elow47 transformants cannot detoxify sufficient amounts of the drug to survive.

When additionally the two *nptII* gene variants were compared, selection for medium-level kanamycin resistance (50 µg mL^−1^) revealed even larger differences. The *EcnptII* gene variant produced only very low numbers of transformed clones and its transformation frequency in strain Elow47 was approximately 50-fold lower than that of *CrnptII* in strain UVM11 (Fig. 7b).

### Antibiotic resistances conferred by *nptII* in *Chlamydomonas*

**Fig. 8:**
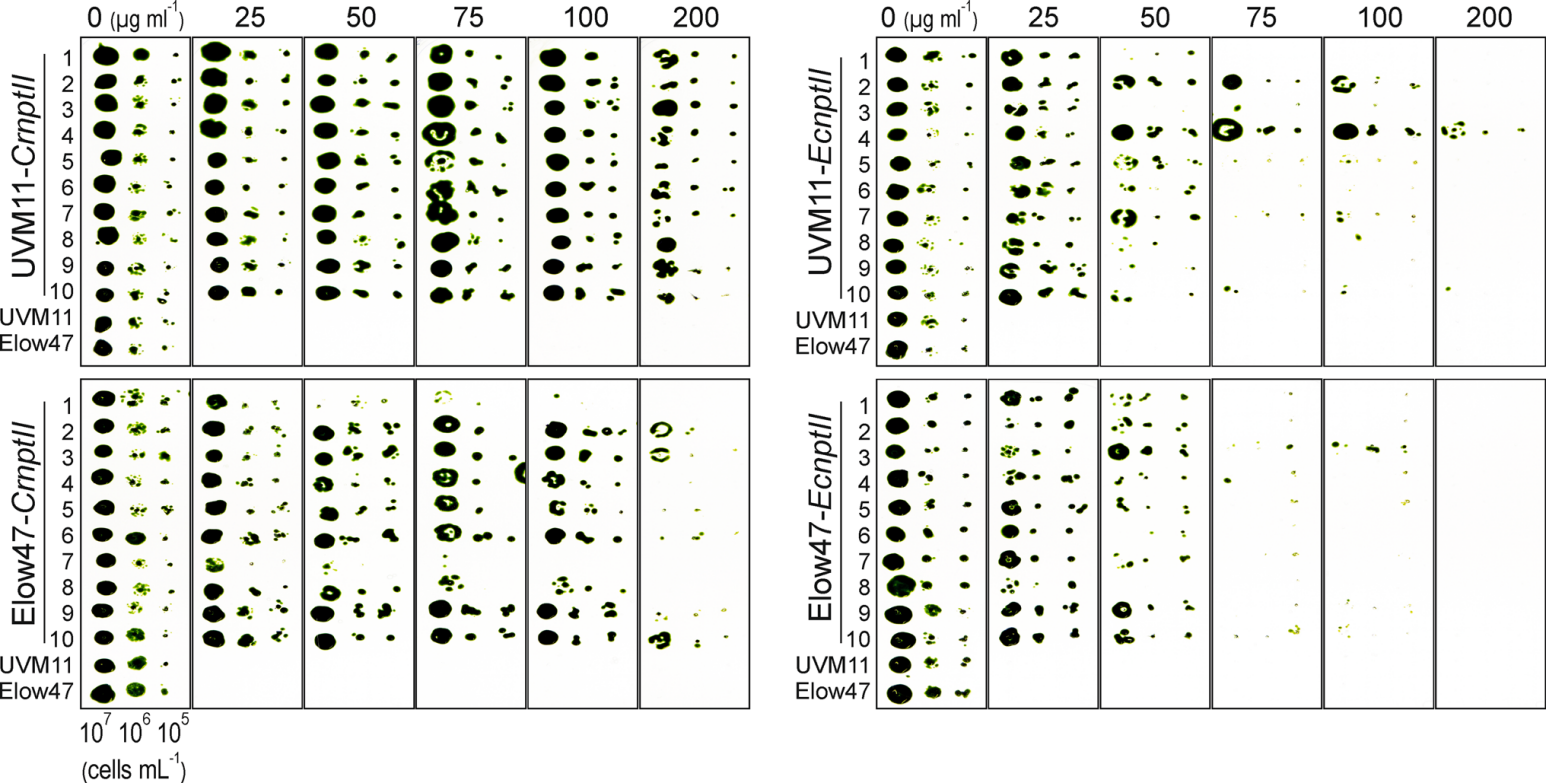
Kanamycin resistance assays with *CrnptII* and *EcnptII* transformants of strains UVM11 and Elow47. The ten transformants per strain and gene variant that had been analyzed with respect to mRNA and protein accumulation levels were assayed for their antibiotic resistance by drop tests with three different cell concentrations (7 µl of cell suspensions with 10^7^, 10^6^ and 10^5^ cells mL^−1^) on agar plates containing different concentrations of kanamycin (0, 25, 50, 75, 100 and 200 µg mL^−1^). Untransformed UVM11 and Elow47 were used as negative controls. Note that all transformed clones shown here were initially selected on kanamycin (50 µg mL^−1^) and, therefore, display some kanamycin resistance. However, on average, *CrnptII* transformants are more resistant to the antibiotic than *EcnptII* transformants, and UVM11 transformants tolerate higher kanamycin concentrations than Elow47 transformants

To test whether NptII expression levels in the two algal strains correlate with the strength of kanamycin resistance, series of drop tests on media with different concentrations of kanamycin (25–200 µg mL^−1^) were performed. Although all tested strains were initially obtained in transformation experiments selecting for resistance to 50 µg mL^−1^ kanamycin, a number of transformants obtained with the *EcnptII* gene and several of the transformants generated in the Elow47 strain grew only poorly in the presence of 50 µg mL^−1^ kanamycin, and some of these even displayed poor growth on 25 µg mL^−1^ in the drop tests (e.g., clones Elow47-*CrnptII*-7; UVM11-*EcnptII*-1 and Elow47-*EcnptII*-8; Fig. 8). By contrast, all transformants with the *CrnptII* gene in expression strain UVM11 showed very strong resistance to kanamycin and all UVM11-*CrnptII* clones continued to grow under the highest antibiotic concentration tested (200 µg mL^−1^). This resistance level was maintained after half a year of strain maintenance under non-selective conditions, confirming that transgene expression in UVM11 is very stable (Barahimipour et al. 2015). As expected from the expression data (Fig. 6), the transgenic clones generated with *EcnptII* in Elow47 grew poorest in the drop tests and all tested transformants died on 200 µg mL^−1^ kanamycin (Fig. 8).

**Fig. 9:**
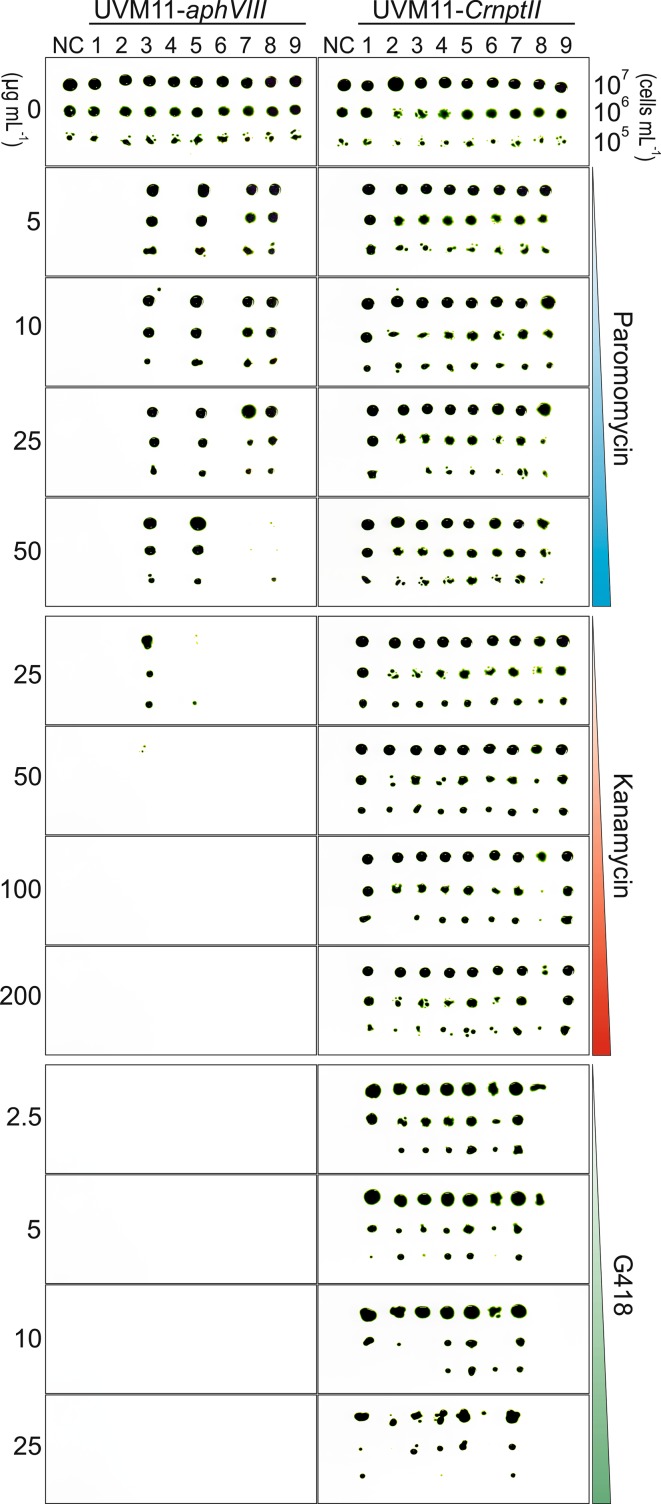
Comparison of the detoxification efficiency of *aphVIII* and *CrnptII* for different antibiotics. Nine randomly selected transformants of UVM11 with either *aphVIII* or *CrnptII* (both initially selected on 25 µg mL^−1^ kanamycin; Sizova et al. 2001) were assayed. The untransformed UVM11 strain was used as negative control (NC). Drop tests were performed using three different cell concentrations (7 µl of cell suspensions containing 10^7^, 10^6^ and 10^5^ cells mL^−1^) on agar plates containing different concentrations of paromomycin (5, 10, 25 and 50 µg mL^−1^), kanamycin (25, 50, 100 and 200 µg mL^−1^) or G418 (2.5, 5, 10 and 25 µg mL^−1^). Photographs were taken after 12 days. Note that, although initially selected on medium containing 25 µg mL^−1^ kanamycin, some *aphVIII* transformants do not grow on kanamycin and paromomycin. This could be due to silencing of the (non-codon-optimized) *aphVIII* transgene during strain maintenance under non-selective conditions

The *nptII* gene encodes the enzyme neomycin phosphotransferase II, an aminoglycoside 3′-phosphotransferase that inactivates, by covalent modification (phosphorylation), a range of aminoglycoside antibiotics, including kanamycin, neomycin, paromomycin and geneticin (G418). To determine whether the optimized *CrnptII* gene in expression strain UVM11 also confers resistance to other aminoglycoside-type antibiotics, we tested UVM11-*CrnptII* transformants on different concentrations of kanamycin, G418 and paromomycin. As an additional control, we produced a set of algal transformants with the paromomycin resistance gene *aphVIII* (overall RCA 61.76 %, GC content 68.9 %, length 804 bp; Fig. 5c), a commonly used selectable marker gene in *Chlamydomonas reinhardtii* (Sizova et al. 2001). *aphVIII* encodes an aminoglycoside phosphotransferase that was shown to not only detoxify paromomycin, but also confer low-level resistance to a few other aminoglycoside drugs, including kanamycin (Sizova et al. 2001). When we compared the kanamycin resistance levels of UVM11-*aphVIII* transformants and UVM11-*CrnptII* transformants (both selected on 25 μg mL^−1^ kanamycin), the *CrnptII* gene turned out to provide much stronger kanamycin resistance than the *aphVIII* gene (Fig. 9). While all UVM11-*CrnptII* transformants grew on 200 µg mL^−1^ kanamycin, only one UVM11-*aphVIII* clone grew reasonably well on 25 µg mL^−1^ kanamycin. Unexpectedly, the UVM11-*CrnptII* transformants also displayed stronger resistance to paromomycin than the UVM11-*aphVIII* transformants (Fig. 9), even though *aphVIII* is the genuine paromomycin resistance gene and the encoded phosphotransferase exhibits its highest substrate affinity towards paromomycin. This finding suggests that, even for paromomycin selection, *CrnptII* outperforms the conventional resistance gene *aphVIII*. When the third aminoglycoside antibiotic, G418 (geneticin), was tested, none of the UVM11-*aphVIII* clones displayed any appreciable resistance (not even on the lowest antibiotic concentration that is required to suppress growth of wild-type cells), indicating that G418 is not a substrate of the AphVIII enzyme, as suspected previously (Sizova et al. 2001). By contrast, eight out of nine UVM11-*CrnptII* transformants showed strong resistance to G418 suggesting that the *CrnptII* marker gene can also be combined with G418 selection. Taken together, these data demonstrate that *CrnptII* represents a new versatile selectable marker for *Chlamydomonas* transformation that facilitates efficient selection of transgenic algal clones and confers strong resistance to at least three different aminoglycoside antibiotics: kanamycin, paromomycin and G418.

## Discussion

In this study, we have investigated two transgenes as case studies for the expression of biotechnologically relevant genes from the nuclear genome of the model green alga *Chlamydomonas reinhardtii*. We selected a protein antigen, the HIV capsid protein P24, and an antibiotic resistance gene (*nptII*) that works as an efficient selectable marker in many prokaryotic and eukaryotic systems, but does not work well in *Chlamydomonas* (Hall et al. 1993; Bingham et al. 1989). As previous work had suggested that (i) codon usage is a major determinant of expression efficiency at the level of the gene sequence (Barahimipour et al. 2015), and (ii) greatly improved expression of fluorescent reporter proteins can be achieved in the UVM4 and UVM11 strains (Neupert et al. 2009; Karcher et al. 2009; Barahimipour et al. 2015), we comparatively analyzed codon-optimized and non-optimized gene variants (encoding the identical amino acid sequence), and tested them in both a UVM expression strain and a wild type-like control strain. Our data demonstrate that, for both transgenes, maximum expression levels are obtained when combining the fully codon-optimized coding region with the UVM11 algal expression strain.

Compared to the non-optimized *P24* gene versions *CpP24* and *P24w*, the non-codon-optimized *nptII* gene (*EcnptII*) performed reasonably well in that, especially in the UVM11 background, it gave rise to detectable gene expression at both the mRNA and the protein levels and also conferred substantial kanamycin resistance (Figs. 6, 7, 8). This is most likely due to the codon usage of the *E. coli nptII* gene (*EcnptII*) being closer to the codon usage in the *Chlamydomonas* nuclear genome than codon usage and GC content of the two non-optimized *P24* gene versions (Figs. 1a, b, 5a, b). The unsuccessful expression of the *CpP24* and *P24w* variants indicates that attempts to express AT-rich transgenes with low relative codon adaptation (RCA) are unlikely to be successful in *Chlamydomonas*.

As noted previously for the *YFP* reporter gene (Barahimipour et al. 2015), mRNA accumulation and protein abundance in the transgenic algal strains are well correlated (e.g., Figs. 2, 3). As transcription of all gene variants is controlled by exactly the same expression cassette and, therefore, transcription rates are expected to be similar, this correlation may suggest that the high translation rates mediated by the fully codon-optimized synthetic gene versions promote mRNA stability. We, therefore, hypothesize, that the difference in mRNA accumulation observed for the different gene variants is due to the different ribosome coverage of the transcripts. This conclusion is in agreement with recent studies in yeast (*Saccharomyces cerevisiae*) that revealed a strong impact of translation rates on mRNA stability (Presnyak et al. 2015). A possible mechanistic explanation could be that, similar to the situation in bacteria, translating ribosomes protect the mRNA from endoribonucleolytic attack by RNA-degrading enzymes (Braun et al. 1998; Sunohara et al. 2004; Deana and Belasco 2005).

In contrast to codon usage and strain background, transgene copy number has no pronounced influence on the attainable expression level (Fig. 4). Thus, the two simple recommendations for optimized transgene expression that can be deduced from this study and previous work (Barahimipour et al. 2015) are to (i) use synthetic genes with fully optimized codon usage for the *Chlamydomonas* nuclear genome, and (ii) introduce the transgenes into a UVM expression strain (Neupert et al. 2009). It is also important to note that, with this strategy, the success rate with transgene expression is close to 100 % in that all transformed UVM11 clones that contained the complete *CrP24* cassette also expressed it to high levels, with only moderate variation in expression strength between independent transformants (Fig. 3). A similar observation was made previously when fluorescent reporter genes were expressed in UVM strains (Neupert et al. 2009; Barahimipour et al. 2015).

Genetic engineering technologies are critically dependent on efficient selection systems for transgenic cells. Although by now, a number of useable selectable marker genes have been established for *Chlamydomonas* (reviewed, e.g., in Weeks 1992; Neupert et al. 2012; Jinkerson and Jonikas 2015; Mussgnug 2015), some of the most efficient markers in other organisms do not work well in *Chlamydomonas*. In this work, we have tested the idea that this is not due to biochemical or physiological peculiarities of *Chlamydomonas* cells (e.g., drug uptake or extrusion problems, rapid drug metabolization or sequestration), but rather to the inefficiency with which foreign genes are expressed in the nucleus of the alga. Taking one of the most widely deployed selectable marker genes, the kanamycin resistance gene *nptII*, as an example, we have shown that this is indeed the case. The codon-optimized *CrnptII* gene variant expressed in expression strain UVM11 allowed efficient selection of transgenic algal clones on kanamycin concentrations that were similarly high (50-75 µg/mL) or even substantially higher than those conventionally used for the selection of transgenic plants (Figs. 7 and 8). Our results explains why, previously, *nptII* could not be established as a selectable marker gene for *Chlamydomonas* transformation (Hall et al., 1993; Bingham et al., 1989). The reason why we obtained a few transformants also with *EcnptII* in the wild type-like strain Elow47 (1, 2 and 7 clones, respectively, in the three independent transformation experiments) may be that we used a stronger promoter to drive the *nptII* gene. The *PSAD* promoter used here controls the expression of an abundant thylakoid protein in the chloroplast (the D subunit of photosystem I) and was shown to be an excellent promoter for transgene expression in *Chlamydomonas* (Fischer and Rochaix 2001).

In our hands, the optimized *nptII* used as selectable marker for transformation of the expression strain is similarly efficient as other antibiotic resistance markers commonly employed for transformation of *Chlamydomonas*, such as hygromycin or paromomycin resistance genes (Berthold et al. 2002; Sizova et al. 2001). Thus, our work provides a new efficient selectable marker gene for *Chlamydomonas* transformation and also suggests a simple strategy for developing additional markers for selection of transgenic algal cells.

As a result of our efforts to optimize expression of the HIV antigen P24 in *Chlamydomonas*, recombinant protein accumulation levels of up to 0.25 % of the total cellular protein were reached (Fig. 3). Previous attempts to express P24 from the tobacco nuclear genome led to accumulation levels of up to 0.35 % of the plant’s total soluble protein (Zhang et al. 2002). Considering that a value expressed as total soluble protein is probably approximately one-third higher than the corresponding value expressed as total cellular protein, the expression levels achieved in tobacco and *Chlamydomonas* are remarkably similar. This suggests that the combination of codon-optimized synthetic genes with our expressions strains largely overcomes the transgene expression problem in *Chlamydomonas* and makes the alga competitive with seed plants as an expression host for recombinant proteins. Although several other algae (e.g., some Chlorella species) can be grown to higher cell densities than *Chlamydomonas*, which is a potential advantage for applications requiring high biomass production, the molecular toolboxes currently available for other algal species do not even come close to that available for *Chlamydomonas*. Also, for many applications, such as the production of high-value biopharmaceuticals, the costs of biomass production are negligible compared the costs for downstream processing and purification which, to a large extent, are determined by the attainable protein accumulation level in the cell (Ma et al., 2005). Finally, we expect that the tools and guidelines for optimized transgene expression, as developed for *Chlamydomonas*, will also be applicable to other algal species.

In summary, the application of the optimization strategies described here will considerably expand the range of biotechnological applications that can be pursued in *Chlamydomonas* and other algae, and likely will facilitate new approaches in metabolic pathway engineering and molecular farming (Scaife et al. 2015).
